# A rare case report of *Salmonella* infection: severe necrotizing pneumonia with empyema in an immunocompetent child

**DOI:** 10.3389/fped.2026.1766995

**Published:** 2026-02-12

**Authors:** Ghofran Waleed Sabbahi, Rayan Abdulsamad Khan, Sultan Saud Altamimi, Maram Mashea Almutairi, Saeed Salem Alghamdi

**Affiliations:** 1 Department of Pediatric, Ministry of Health, Jeddah, Saudi Arabia; 2 Department of General Pediatric, Al Aziziyah Children Hospital, Jeddah, Saudi Arabia; 3 Department of Pediatric Intensive Care Unit, Ministry of Health, Jeddah, Saudi Arabia; 4 Section of Pediatric Intensive Care, International Medical Center, Jeddah, Saudi Arabia

**Keywords:** cytokine storm, extracorporeal membrane oxygenation, immunocompetent child, influenzacoinfection, necrotizing pneumonia, pediatric empyema, *salmonella* infection, severepneumonia

## Abstract

*Salmonella* is a gram-negative bacillus that typically causes gastrointestinal disease and rarely affects the pulmonary system, particularly in immunocompetent children. We report a 4-year-old immunocompetent Saudi boy who presented with severe respiratory distress requiring intubation and was found to have left-sided pleural effusion. Pleural fluid and tracheal aspirate cultures grew *Salmonella* species, while blood, stool, and CSF cultures were negative. Chest computed tomography revealed complicated empyema with necrotizing pneumonia. Despite appropriate antimicrobial therapy and supportive care, the patient deteriorated and ultimately required extracorporeal membrane oxygenation (ECMO). This case illustrates a rare and severe presentation of complicated pulmonary *Salmonella* infection in an otherwise healthy child, occurring either following or concurrent with influenza infection. It underscores the critical importance of timely source control and highlights the potential association with cytokine storm in severe disease progression.

## 1 Introduction

*Salmonella* species are motile, gram-negative, hydrogen-sulfide–producing bacilli that primarily infect the gastrointestinal tract and are well-known causes of gastroenteritis worldwide, particularly in developing countries. Transmission is commonly associated with ingestion of contaminated water or food of animal origin (1, 2). The Institute for Health Metrics and Evaluation (IHME) estimated that in 2019 there were 594,000 cases of invasive non-typhoidal *Salmonella* disease (iNTS) globally, resulting in 79,000 deaths and 6.11 million Disability-Adjusted Life Years (DALYs), with an all-age case fatality rate of 14.5%, and up to 41.8% among people living with HIV. In contrast, diarrheal non-typhoidal *Salmonella* (dNTS) is more prevalent and usually self-limiting, yet in 2019 it was estimated to cause 73.9 million cases and 61,600 deaths worldwide, with a substantial burden in children under five (3). In Saudi Arabia, *Salmonella* remains a significant cause of food-borne disease, with non-typhoidal *Salmonella* (NTS) accounting for most pediatric bacterial gastroenteritis cases (4–6). Although national incidence varies, one study reported an average incidence of 6.04 per 100,000 children under 15 years of age between 2013 and 2017 (5). According to the most recent Saudi Ministry of Health statistical yearbook, a total of 2,286 NTS cases were reported in 2024, with the highest incidence observed in children under one year and adults aged 15–45 years. This corresponds to an incidence rate of 6.48 per 100,000 population, up from 4.48 per 100,000 in 2022, reflecting an increasing trend (7). Although national data on mortality and economic burden are not publicly available, global evidence indicates that NTS imposes substantial direct medical costs and productivity losses, and remains a significant cause of morbidity and mortality, particularly among high-risk populations. Extraintestinal salmonellosis, including bacteremia, central nervous system infection, and osteoarticular infection, is uncommon (2, 6). Pulmonary involvement is exceedingly rare, especially in immunocompetent children, with only isolated cases reported in the literature (1, 2, 5). In this case report, we describe a child presenting with fever and respiratory distress who was found to have severe complicated pneumonia secondary to isolated *Salmonella* recovered from the lower respiratory tract.

## 2 Case description

A 4-year-old Saudi boy, previously healthy, fully vaccinated for age, and with no history suggesting primary immunodeficiency, sickle cell disease, or other risk factors for invasive *Salmonella* infection, presented to the emergency department with a 12-day history of fever reaching 40°C (measured axillary at home), associated with cough and coryzal symptoms. His symptoms progressively worsened, and three days prior to presentation he developed increasing shortness of breath. There were no gastrointestinal symptoms and no recent travel. The only significant history was exposure to sick family members who have gastrointestinal symptoms.

On examination, the patient appeared ill, febrile (38.6°C), and in severe respiratory distress. He was hypoxemic (SaO_2_ 85% on room air) and tachypneic (respiratory rate 45/min) with subcostal and intercostal retractions, grunting, and markedly decreased air entry over the left lung. Cardiovascular examination was normal, and the abdomen was soft and non-tender with no hepatosplenomegaly. There was no rash or lymphadenopathy. Due to worsening respiratory failure, he was emergently intubated and placed on mechanical ventilation.

**Figure 1:**
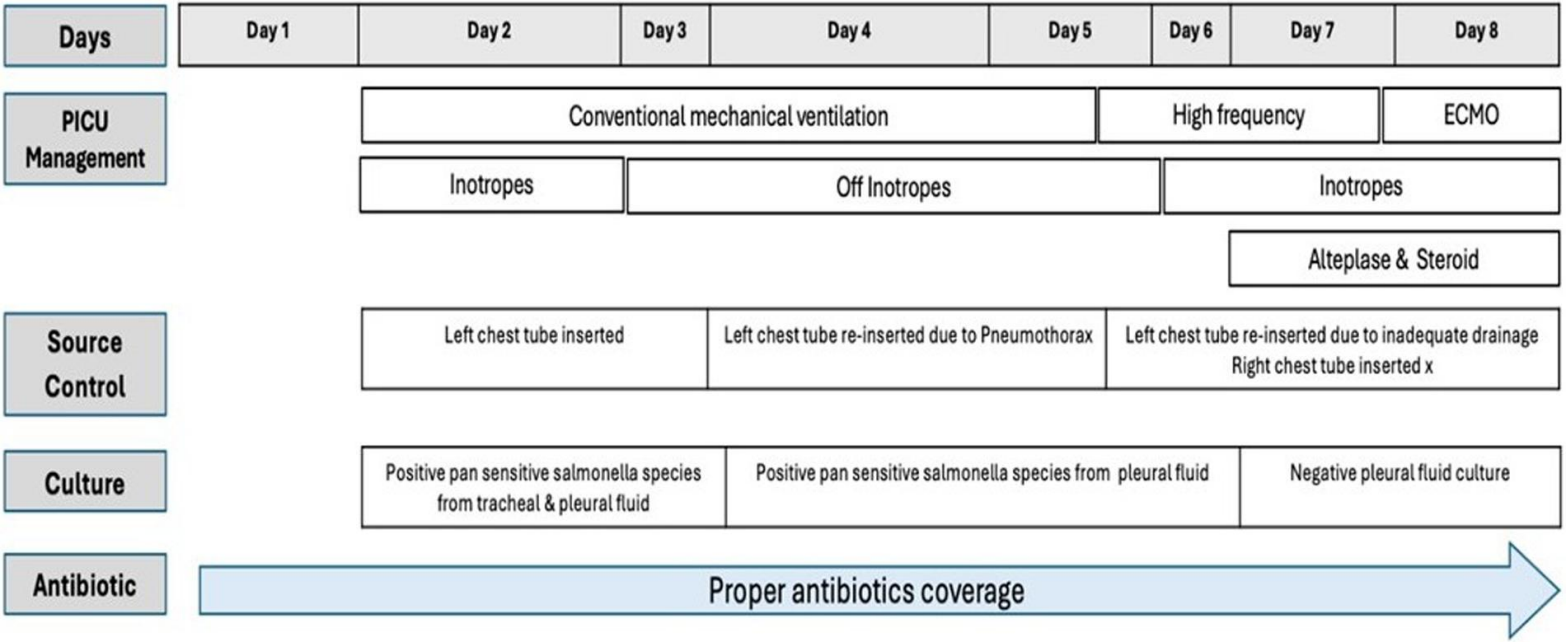
Patient's clinical course timeline.

Initial laboratory investigations showed a normal complete blood count, acute kidney injury (creatinine 1.04 mg/dL; BUN 50 mg/dL), and markedly elevated C-reactive protein (90 mg/L). Chest radiography demonstrated a left-sided pleural effusion (Figure 1). A left-sided chest tube was placed, draining approximately 30 mL of black fluid, which remained consistently black in subsequent drainage. It was sent for chemistry, cytology, and microbiology. Pleural fluid analysis revealed 253 cells/mm^3^ (70% polymorphs, 30% lymphocytes) and protein level of 2,267 mg/dL, consistent with an exudate. Empiric intravenous ceftriaxone and linezolid were initiated. Gram stain showed gram-negative bacilli. After 48 h, cultures from pleural fluid and tracheal aspirate grew *Salmonella* species, sensitive to ceftriaxone, ceftazidime, meropenem, and ciprofloxacin. Respiratory viral panel was positive for Influenza A. The patient was continued on ceftriaxone. Blood, cerebrospinal fluid, urine, and stool cultures were negative. Immunological evaluation including HIV testing, IgG/IgM/IgA levels, and lymphocyte subset flow cytometry were all normal for the child's age.

**Figure 2:**
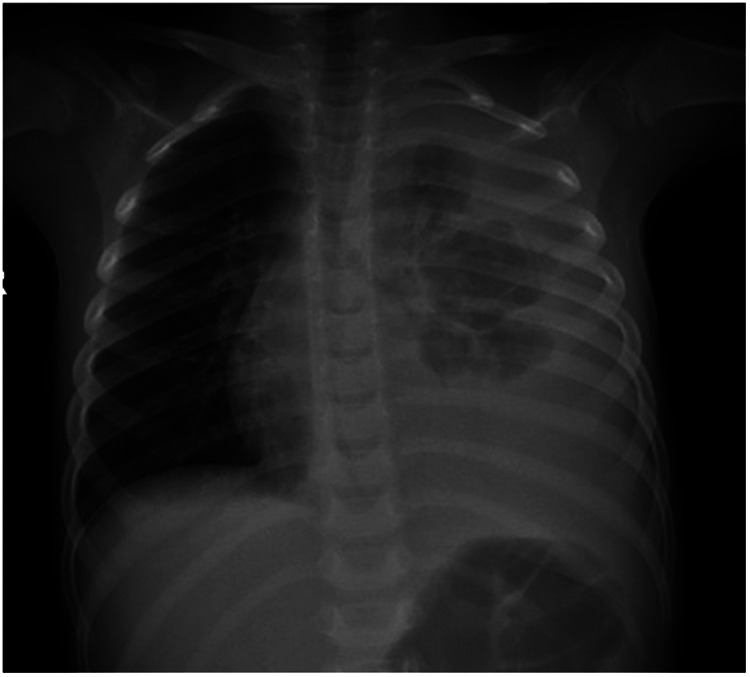
Moderate to severe left plural effusion. Left lung shows multiple round homogonous increased opacification occupying left lower zone and sharply marginated superiorly.

Over the next few days, the patient's renal function normalized; however, his respiratory status progressively deteriorated. He developed acute respiratory distress syndrome (ARDS), with worsening hypoxemia and a declining PaO_2_/FiO_2_ ratio (<100), bilateral infiltrates on imaging, and respiratory failure unexplained by cardiac disease. He subsequently developed a new right-sided pleural effusion and a left upper pneumothorax. Contrast enhanced CT of the chest revealed extensive bilateral consolidations and loculated pleural collections, consistent with necrotizing pneumonia complicated with empyema, without evidence of a definite bronchopleural fistula (Figure 2).

The left chest tube required multiple revisions, with a total of three replacements due to pneumothorax, persistent loculations, and inadequate drainage. A right-sided chest tube was inserted and drained sterile fluid. Although the child had multiloculated empyema, intrapleural alteplase therapy was initiated late and was only administered later through the left chest tube.

During this period, repeated cultures from pleural fluid obtained via the left-sided chest tube remained persistently positive, yielding the same isolate with an identical antimicrobial susceptibility test results. Following the third chest tube revision, concerns were raised regarding a possible emerging resistant organism in the context of ongoing left-sided pleural fluid drainage so cultures were repeated and antibiotics escalated. In parallel, the development of a cytokine-mediated inflammatory syndrome was considered, given the child's continued clinical deterioration despite appropriate antimicrobial therapy, radiologic progression, and persistent pleural effusion. This concern was supported by laboratory findings showing hemoglobin 8.4 g/dL, white blood cells 4.0 × 10⁹/L, absolute neutrophil count 3.4 × 10⁹/L, absolute lymphocyte count 0.6 × 10⁹/L, platelets 113 × 10⁹/L, CRP 270 mg/L, ferritin 351 ng/mL, Lactate Dehydrogenase 860 U/L, and fibrinogen 7.0 g/L Aspartate Aminotransferase 344 U/L, Alanine Aminotransferase 61 U/L, Albumin 3.3 g/dL, INR 1.38 s PT 18.5 s PTT 37.5 s

According to the patient's clinical status at that time and given laboratory result, antimicrobial therapy was escalated to meropenem, and vancomycin was added while awaiting the results of repeat cultures. In addition, high-dose corticosteroid therapy was initiated for suspected cytokine storm syndrome, and a single dose of intravenous immunoglobulin (IVIG) was administered due to concern for a possible primary immunodeficiency.

Unfortunately, the child's clinical course was complicated by progressive hypoxemia with worsening PaO₂/FiO₂ ratios, ultimately failing conventional mechanical ventilation. Despite escalation to high-frequency oscillatory ventilation, respiratory failure persisted, necessitating initiation of veno-venous extracorporeal membrane oxygenation (VV-ECMO) and transfer to a tertiary care center with ECMO capability.

Following stabilization on VV-ECMO, the patient demonstrated gradual respiratory improvement and was successfully weaned back to conventional mechanical ventilation. As his pulmonary status improved and pleural drainage decreased, chest tubes were removed sequentially, one at a time, without complications. Antimicrobial therapy was reassessed during this period; vancomycin was discontinued, while meropenem was continued despite repeatedly negative cultures from blood, pleural fluid, and endotracheal aspirates.

The patient was subsequently extubated and transferred to the pediatric medical ward, where he required non-invasive oxygen support via nasal cannula for an additional two weeks. He completed a total of six weeks of antimicrobial therapy with good clinical response.

Ultimately, after a prolonged and complex clinical course, the child survived and was discharged home in good condition. Follow-up communication with the family confirmed that he remained clinically well and in good health at the time of discharge.

**Figure 3:**
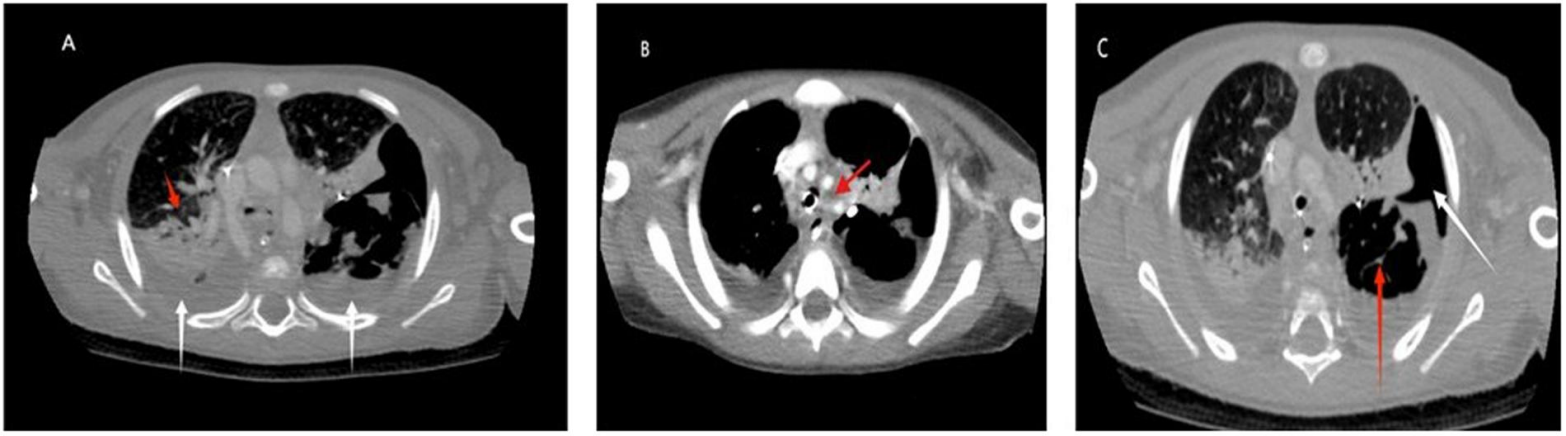
**(A)** Red arrow: air bronchogram seen in the right upper lobe with scattered surrounded rounded nodular consolidations and ground glass densities. White arrow: bilateral small to medium-sized pleural effusion with adjacent lung atelectasis. **(B)** There are associated multiple enlarged mediastinal, hilar and axillary lymph nodes. Some of them are necrotic (in red arrow). **(C)** Red arrow: The left lower lobe demonstrates internal necrotic components with multiple air pockets and internal septations suggestive of necrotizing infection. White arrow: hydropneumothorax on the left lung superiorly as a complication.

The patient's clinical course and major interventions are summarized in Figure 3.

## 3 Discussion

This case represents an exceptionally rare and complex clinical scenario of complicated pneumonia by empyema and necrotizing pneumonia, because it involves the coexistence of *Salmonella* infection and influenza an association that, to our knowledge, has not been previously described in the literature, especially among pediatric patients and in Saudi Arabia.

A review of the literature revealed approximately nine reported cases of *Salmonella*-complicated pneumonia worldwide among the pediatric age group. To our knowledge, our case represents the first pediatric reported instance in Saudi Arabia. Comparing our case with previous reports, most patients were immunocompetent, except for the case published in Turkey, where the patient had an underlying hemoglobinopathy (8). In addition, in case reported by Edward E Eaton et al., in USA had HIV positive (9). Another reported cases involved patients with chronic lung infection, such as hydatid cysts or tuberculosis, as described by Aslam et al., who reported a case of *Salmonella* Typhi infection in a lung hydatid cyst (10).

Influenza coinfection, particularly during peak seasonal activity, is a well-recognized leading cause of severe lower respiratory tract infection as it predisposes patients to secondary bacterial pneumonia. This phenomenon is classically described with *Streptococcus pneumoniae*, *Staphylococcus aureus*, and Group A *Streptococcus*. However, in our patient, influenza appears to have been the main predisposing factor leading to the development of complicated pneumonia. Previous reports have described *Salmonella* empyema in association with underlying structural lung infection such as hydatid cysts and tuberculosis; similarly, influenza may represent an important predisposing insult in this case (10).

Although necrotizing pneumonia is classically associated with Gram positive organisms such as *Staphylococcus aureus* and *Streptococcus pneumoniae*, our case demonstrates that, although rare, *Salmonella* spp. can also serve as a causative agent even in an immunocompetent child. Notably, all blood cultures were negative, including repeated samples. This is consistent with existing literature review showing that fewer than 10% of necrotizing pneumonia cases yield positive blood cultures. In contrast, pleural fluid cultures have a significantly higher yield, and more than 90% of empyema pathogens can be isolated from the pleural space as the infection progresses (11).

Another unique aspect of this case was the black discoloration of the pleural fluid. This finding can be correlated with CT evidence of necrotizing pneumonia, and pleural fluid analysis which demonstrated red blood cells of 6,764, supporting ongoing hemorrhagic process. Such hemorrhagic pleural involvement is consistent with severe parenchymal destruction extending into the pleura secondary to necrotizing pneumonia (12).

Black pleural fluid is a rare finding with limited reported causes, broadly classified as infectious or non-infectious. Infectious etiologies are mainly fungal, particularly *Aspergillus niger* and *Rhizopus oryzae*, and it has also been reported in patients with underlying pulmonary disease, such as COPD or tuberculosis. Non-infectious causes include malignancy and autoimmune disorders like SLE (12). In our patient, clinical evaluation, negative atypical cells in pleural fluid, autoimmune workup, and a negative TB workup make all these possibilities less likely.

**Figure 4:**
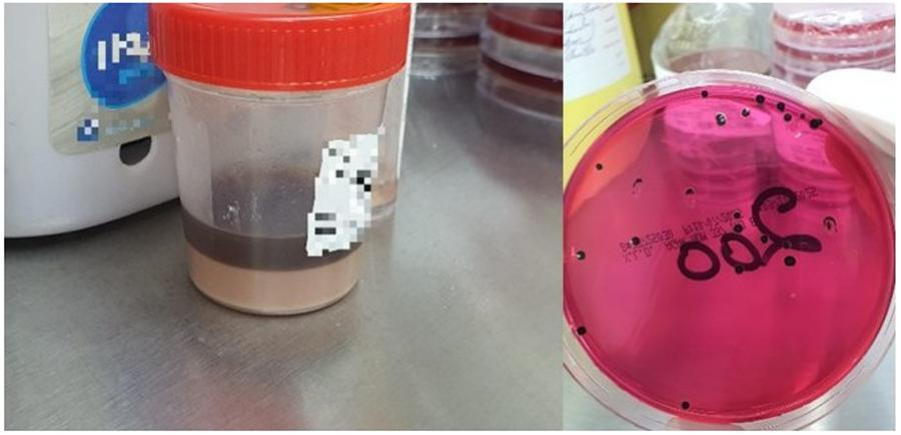
Body fluid appearance and black dots on culture agar due to hydrogen sulfide produced by salmonella.

The persistent black discoloration of the pleural fluid during the early phase of illness was most likely attributable to *Salmonella* infection in the context of progressive necrotizing pneumonia and inadequate source control, as evidenced by the frequent need for chest tube replacement and ongoing clinical deterioration. *Salmonella* species are known producers of hydrogen sulfide (H_2_S), a feature that results in the characteristic blackening observed on selective media such as XLD. In these media, H₂S reacts with iron to generate dark iron sulfide precipitates, producing the black centered colonies. We assume that a similar biochemical process may have occurred within the pleural space. In the setting of necrotizing infection, severe parenchymal lung destruction likely resulted in damage to local blood vessels, allowing red blood cells to leak into the pleural space. Concurrently, hydrogen sulfide (H_2_S) produced by *Salmonella* may have interacted with iron-containing components within the pleural fluid, contributing to the black discoloration observed in our patient. This hypothesis is supported by both the gross appearance of the pleural fluid and the characteristic black colonies seen on the XLD culture plate (Figure 4).

The pathogenesis of invasive bacterial infection in our case remains unclear, similar to most previously reported cases, as all blood and stool cultures were negative. This suggests that the infection was primarily localized to the lungs, likely aggravated by specific contributing factors; such as transient or disseminated bacteremia, underlying primary or secondary immunodeficiency, hemoglobinopathies, and structural lung abnormalities resulting from congenital conditions or chronic pulmonary infections. However, in our case, this may be related to ongoing necrotizing pneumonia, presumably secondary to a preceding influenza infection.

Several mechanisms may explain the negative blood cultures. Prior antibiotic exposure can reduce bacterial yield, leading to false-negative results. Another explanation is transient translocation across the intestinal mucosa without sustained bacteremia, allowing the organism to seed distant sites while remaining undetectable in the bloodstream; however, repeated cultures in our patient remained negative. A further possibility is bacterial localization within gastrointestinal lymphatic pathways, permitting migration to the pleural space via lymphatic channels, which could represent the actual mechanism in our patient, especially if we assume his immunity was intact and considering that the prior influenza infection may have weakened the pulmonary tissue (11).

In addition to host-related factors, environmental and exposure-related factors must also be considered, particularly in pediatric patients. Non-typhoidal *Salmonella* (NTS) infections are commonly associated with poor hygiene, consumption of undercooked meat, unpasteurized milk or dairy products, and person-to-person transmission via the fecal–oral route. Notably, the child had a history of close household exposure, as his brother developed gastrointestinal symptoms shortly before the onset of the patient's illness, raising the possibility of asymptomatic carriage or intrafamilial transmission despite negative stool cultures. Although a definitive exposure source could not be confirmed, such factors remain important contributors to sporadic pediatric NTS infections and should be considered when assessing risk in children.

In our case, we believe the patient developed severe complicated pneumonia due to *Salmonella* empyema, likely triggered by a preceding influenza infection that progressed to necrotizing pneumonia. Assuming the patient is immunocompetent and has no underlying chronic granulomatous disease (CGD) which could not be confirmed due to institutional limitations the rapid clinical deterioration, despite timely and appropriate antimicrobial therapy and the eventual need for ECMO, can be explained by several factors. Inadequate source control was evident, as there was a delay in initiating fibrinolytic therapy, and frequent chest tube replacements limited effective drainage, resulting in persistent pleural fluid accumulation and progressive clinical decline. Additionally, the patient developed a cytokine storm, manifested by cytopenia, hepatosplenomegaly that appeared in the later days, and coagulopathy, raising concern for macrophage activation syndrome (MAS) or hemophagocytic lymphohistiocytosis (HLH). Common predisposing factors for severe non-typhoidal *Salmonella* infection, including hemoglobinopathies, HIV, gastrointestinal abnormalities, and chronic use of proton pump inhibitors, were excluded, leaving the possibility of an underlying primary immunodeficiency, such as CGD, Toll-pathway defects, or other immune-mediated disorders, which we were unable to investigate at our institution due to resource limitations, and which may explain the unusually severe disease course observed in this patient.

In addition to host-related factors, environmental and exposure-related factors must also be considered, particularly in pediatric patients. Non-typhoidal *Salmonella* (NTS) infections are commonly associated with poor hygiene, consumption of undercooked meat, unpasteurized milk or dairy products, and person-to-person transmission via the fecal–oral route. Notably, the child had a history of close household exposure, as his brother developed gastrointestinal symptoms shortly before the onset of the patient's illness, raising the possibility of asymptomatic carriage or intrafamilial transmission despite negative stool cultures. Although a definitive exposure source could not be confirmed, such factors remain important contributors to sporadic pediatric NTS infections and should be considered when assessing risk in children.

In our setting, *Salmonella* serotyping and molecular testing were not available at the time of diagnosis, preventing detailed serovar identification. Moreover, the negative stool culture observed in our patient is likely attributable to intermittent fecal shedding of non-typhoidal *Salmonella*, which may decrease as the disease progresses, as well as prior exposure to antibiotics before sample collection. Negative stool cultures in invasive *Salmonella* infections, including *Salmonella* empyema, have been well documented despite evidence of ongoing systemic infection. In our case, this can be explained by the combination of intermittent bacterial shedding, timing of specimen collection relative to the course of illness, and prior antibiotic exposure.

These diagnostic limitations, together with the negative stool culture, underscore the need for improved access to advanced diagnostic modalities, which can enhance diagnostic yield, facilitate epidemiological tracking, and strengthen food safety surveillance systems. The absence of such tools limits the ability to link clinical isolates to potential foodborne sources, trace transmission pathways, and contribute meaningfully to broader epidemiological surveillance, all of which are essential for effective public health monitoring and prevention in our region and worldwide.

## 4 Conclusion

This case represents a rare and severe instance of complicated pulmonary Salmonella empyema and necrotizing pneumonia in an otherwise healthy child, occurring either following or concurrent with influenza infection. It highlights the critical importance of timely source control and the potential role of cytokine storm in severe disease progression. Furthermore, it underscores the need for preventive measures against non-typhoidal *Salmonella* (NTS) infections, including adherence to global food safety standards, such as proper cooking of meat, pasteurization of dairy products, safe food handling, and good hygiene practices. Public health interventions that improve food safety and reduce exposure are essential, particularly for vulnerable populations such as young children and immunocompromised patients. Finally, we emphasize the need for further research in our region to accurately assess the economic burden and mortality associated with NTS, as current national data are limited. Such studies are crucial to guide effective public health strategies and resource allocation for the prevention and management of NTS infections.

## Data Availability

The original contributions presented in the study are included in the article/Supplementary Material, further inquiries can be directed to the corresponding author.
